# Transglutaminase‐2 Promotes Microglial Synaptic Phagocytosis and Ameliorates Epileptic Seizures by Inhibiting ABCA1 Ubiquitination

**DOI:** 10.1002/cns.70725

**Published:** 2025-12-28

**Authors:** Zunlin Zhou, Xiujuan Wang, Juan Yang, Jiyao Qin, Bidan Feng, Qianqiong Qin, Jun Tian, Zhong Luo, Xiaoyan Yang, Hao Huang, Xin Xu, Juan Li, Zucai Xu, Changyin Yu, Haiqing Zhang

**Affiliations:** ^1^ Department of Neurology Key Laboratory of Brain Function and Brain Disease Prevention and Treatment of Guizhou Province (ZSYS(2025)030), Affiliated Hospital of Zunyi Medical University Zunyi Guizhou China; ^2^ Department of Neurology Chongqing Key Laboratory of Neurology, First Affiliated Hospital of Chongqing Medical University Chongqing China

## Abstract

**Background:**

Epilepsy is a prevalent chronic neurological disorder characterized by its complex pathophysiology, with microglial phagocytosis being crucial for synaptic remodeling and epileptogenesis. Transglutaminase‐2 (TGM2) holds a critical role in regulating microglial function and cognitive synaptic plasticity; however, the precise mechanisms by which TGM2 influences synaptic pruning and epileptogenesis remain unclear.

**Aim:**

This study aims to investigate the role of TGM2 in seizure susceptibility and its regulatory effects on microglial‐mediated synaptic phagocytosis in a chronic epilepsy model. Accordingly, the following objectives were set: elucidate the fluorescent localization and protein expression characteristics of TGM2 in normal and epileptic brain tissues; analyze the impact of TGM2 on epileptic behavioral phenotypes; and investigate the molecular mechanisms underlying its regulation of microglial activation and synaptic phagocytic function using an epileptic mouse model.

**Methods:**

In vivo experiments were performed using a kainic acid (KA)–induced chronic epilepsy mouse model established via intrahippocampal injection. Western blot and immunofluorescence analyses were employed to examine TGM2 expression and localization in the hippocampus of KA‐treated mice. Adeno‐associated virus vectors were used to achieve TGM2 overexpression or knockdown in the hippocampus, after which video‐monitored behavioral assays and in vivo field potential recordings were used to evaluate seizure latency, frequency, and severity. Golgi–Cox staining, western blotting, and immunofluorescence were used to assess dendritic spine density in the hippocampal CA1 region, microglial polarization (M1/M2 phenotypes), and phagocytic activity. In vitro studies in BV2 microglia explored the molecular mechanisms of action of TGM2 using ubiquitination assays targeting ATP‐binding cassette transporter A1 (ABCA1).

**Results:**

TGM2 expression was significantly upregulated in the hippocampus of KA‐induced epileptic mice, which prolonged the latency period to spontaneous recurrent seizures (SRS) and reduced SRS frequency. In contrast, TGM2 knockdown exacerbated seizure severity, which was characterized by a shortened latency period and increased SRS frequency. Golgi–Cox staining revealed that TGM2 overexpression decreased dendritic spine density in the CA1 region, whereas TGM2 knockdown had the opposite effect, indicating a role in synaptic remodeling. Functional analyses showed that TGM2 promoted microglial polarization toward an anti‐inflammatory M2 phenotype, enhanced phagocytic activity, and upregulated the components of the complement system as well as the phagocytosis‐related proteins. Conversely, TGM2 deficiency promoted the pro‐inflammatory M1 phenotype, reduced phagocytic capacity, and downregulated the components of the complement system and the phagocytosis‐related proteins. Mechanistically, TGM2 overexpression increased ABCA1 protein stability by inhibiting its ubiquitination, whereas TGM2 knockdown promoted ABCA1 ubiquitination and degradation. Immunofluorescence analysis revealed enhanced colocalization of TGM2 within the microglia.

**Conclusion:**

This study revealed that TGM2 suppresses epileptogenesis by enhancing microglial synaptic phagocytosis through the inhibition of ABCA1 ubiquitination, thereby regulating synaptic remodeling in the hippocampus. These findings establish a critical molecular link between TGM2‐mediated microglial function and epileptogenesis, providing novel insights into therapeutic strategies targeting neuroinflammation and synaptic plasticity in epilepsy.

## Graphical abstract

Our study reveals that Transglutaminase‐2 functions as a key endogenous suppressor of seizure susceptibility. It mediates this effect by promoting microglial phagocytosis of synapses through the inhibition of ABCA1 ubiquitination and subsequent increase in its protein stability. This identifies a novel molecular pathway where TGM2 links microglial function to synaptic remodeling in epilepsy, offering a new potential therapeutic target.

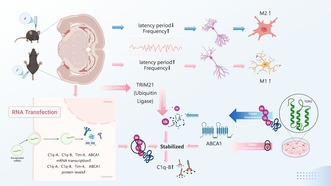

## 1 Introduction

Epilepsy is a common neurological disorder characterized by abnormal brain discharge, resulting in spontaneous recurrent seizures (SRS) [1]. Epilepsy affects approximately 0.4%–1.0% of the global population [2]. Compared with healthy individuals, patients with epilepsy are more likely to experience comorbidity [3, 4], psychological dysfunction [5], social discrimination, and economic burden, which substantially influence their daily quality of life. Approximately 30 anti‐seizure medications are available for treating various types of epilepsy; however, nearly 30% of patients remain refractory to treatment [6, 7]. Treating epilepsy is often challenging, partly because the underlying mechanisms of epileptogenesis following the initial injury are uncertain.

Microglia are resident macrophages in the central nervous system (CNS); their functions include surveillance of the CNS parenchyma, removal of abnormal components, and phagocytosis to regulate synaptic homeostasis [8, 9]. Microglial phagocytosis helps maintain the delicate balance of the neural network by recognizing, engulfing, and degrading neuronal fragments, abnormal protein aggregates, and redundant synaptic structures [10]. Recent investigations have demonstrated that microglial phagocytic activity is mechanistically implicated in the pathogenesis of epilepsy through modulation of synaptic remodeling [11, 12, 13]. The stability of synaptic functions relies on the dynamic equilibrium between synaptogenesis and clearance, with microglia playing a pivotal role in synaptic pruning via phagocytosis [12]. Under physiological states, microglia selectively eliminate defective or redundant synapses to optimize the information transfer efficiency in neural circuits [9]. However, under epileptic conditions, the phagocytic activity of microglia becomes dysregulated: excessive phagocytosis induces synaptic over‐loss, disrupting inhibitory neural circuits and exacerbating neuronal excitatory imbalance; conversely, insufficient phagocytosis allows persistent retention of hyperexcitable or immature synaptic structures, facilitating the formation of aberrant neural networks [9, 12]. Following a seizure, the brain microenvironment undergoes substantial changes, including the substantial release of excitatory neurotransmitters, elevation of inflammatory factors, and heightened oxidative stress [14, 15]. These pathological signals activate microglia and trigger modification of their phagocytic profiles via modulation of surface receptors and downstream signaling pathways [16, 17].

Transglutaminase‐2 (TGM2), a multifunctional calcium‐dependent enzyme, is widely expressed in the CNS, immune cells, and peripheral tissues [18, 19]. The canonical activity of TGM2 involves the catalysis of covalent cross‐linking between glutamine and lysine residues in proteins, which in turn regulates extracellular matrix (ECM) stability, cell signaling, and apoptosis [19, 20, 21]. Pathologically, TGM2 dysfunction is linked to diverse diseases: it promotes tumor invasion and drug resistance in cancer [22, 23], while mediating aberrant protein aggregation and neuroinflammation in Alzheimer's and Huntington's diseases [24]. Physiologically, TGM2 strengthens tissue mechanical properties by cross‐linking ECM components (e.g., fibronectin and collagen); it also modulates cell survival and DNA repair. In the nervous system, TGM2 regulates chromatin accessibility as well as the expression of genes related to synaptic plasticity via histone monoamidation [25, 26, 27].

TGM2 is critical for microglial activation and functional regulation, governing synaptic recognition and phagocytosis. TGM2 activity is upregulated under inflammatory conditions, prompting microglia to recognize and eliminate redundant or abnormal synaptic connections and thus facilitating targeted phagocytosis by labeling specific synaptic surface molecules [28]. An imbalance in microglial synaptic phagocytosis—characterized by the excessive elimination of inhibitory synapses or the retention of aberrant excitatory connections—can trigger abnormal neuronal hyperexcitability and hypersynchronous discharge. This mechanism is particularly evident in epilepsy, where synaptic plasticity dysregulation intersects with aberrant microglial activation, collectively exacerbating pathological excitation in neural networks [9, 29]. These findings indicate that TGM2 modulates neuronal network excitability by regulating microglial synaptic remodeling, positioning it as a potential key regulator of excitation–inhibition imbalance in epileptogenesis. However, the roles and mechanisms of TGM2 in microglial‐mediated synaptic phagocytosis, as well as its contribution to sustained neuronal excitability in epilepsy, remain understudied.

In light of the current evidence, we propose that TGM2 modulates synaptic plasticity by mediating microglial phagocytosis during epileptogenesis. To investigate the role of TGM2 in epilepsy, we evaluated its expression changes and functional implications using both in vitro and in vivo models. Our evidence demonstrates that increased TGM2 expression—observed primarily in microglia—exerts a protective effect by inducing microglial‐mediated synaptic pruning, promoting microglial polarization toward the M2 phenotype, and reducing seizure occurrence in mice. Mechanistically, TGM2 overexpression decreased ubiquitination of ATP‐binding cassette subfamily A member 1 (ABCA1)—a key regulator of lipid homeostasis and phagocytic activity. Collectively, these findings establish TGM2 as a critical protective factor against epilepsy, highlighting its potential as a therapeutic target for intervention.

## 2 Materials and Methods

### 2.1 Animals

Male C57BL/6 mice (aged 7–8 weeks, weighing 20–25 g) were bred and maintained under standardized conditions at the Laboratory Animal Center of Chongqing Medical University. Experimental procedures were approved by the Ethical Review Committee for Laboratory Animals of Chongqing Medical University (Ethical Approval Number: IACUC‐CQMU‐2024‐0277) and adhered to the International Guidelines for Laboratory Animal Welfare (AAALAC standards) and relevant Chinese regulations on laboratory animal management. The study followed the ARRIVE (Animal Research: Reporting of In Vivo Experiments) Guidelines 2.0, and measures were implemented to reduce the number of animals used and mitigate animal suffering. Animals were housed in a specific pathogen‐free facility equipped with an environmental control system. Standardized husbandry protocols were strictly followed. A maximum of five mice were housed per cage under an automated 12‐h light/dark cycle at 22°C ± 1°C. Access to irradiated standard rodent chow and autoclaved drinking water was provided ad libitum.

### 2.2 Virus Construction and Hippocampal Injection

Recombinant adeno‐associated viruses (AAVs) were constructed by Obio Technology Corp. Ltd. (Shanghai, China). These viral vectors incorporated a green fluorescent protein (GFP) reporter gene system for real‐time monitoring of transfection efficiency. The vector PCAAV‐CMV‐Tgm2‐3xFLAG‐P2A‐EGFP‐tWPA (Gene ID: 21817; abbreviated as AAV‐Tgm2) was engineered to mediate *TGM2* overexpression. The vector pAAV‐U6‐shRNA (Tgm2)‐CMV‐EGFP‐WPRE (abbreviated as shTgm2)—carrying the shRNA‐targeting mouse *TGM2* sequence 5′‐CGCTTCTCACTGTCTGACAAT‐3′—was created to achieve *TGM2* silencing. Con‐AAV‐Tgm2 (PCAAV‐CMV‐EGFP‐tWPA) and Con‐shTgm2 (pAAV‐U6‐shRNA(NC)‐CMV‐EGFP‐WPRE) were constructed in parallel as blank control vectors.

To achieve specific viral expression in the CA1 and dentate gyrus (DG) subregions of the hippocampus, we employed a validated single‐needle dual‐site stereotaxic injection strategy [30]. Mice were anesthetized and fixed in a stereotaxic apparatus. The skull was exposed and the Bregma point was identified. Based on the mouse brain atlas, coordinates for the DG region were calculated (Bregma −1.9 mm, left of the fornix +1.2 mm), and a cranial burr hole was drilled at this point. Subsequently, a microinjection cannula (Hamilton, Reno, USA) was vertically advanced to a depth of −2.2 mm along the dorso–ventral axis, and 1.2 μL of the respective AAV was injected at a slow rate of 100 nL/min. After injection, the needle was left in place for 5 min to prevent viral backflow along the needle track. Subsequently, the needle was slowly advanced to the target depth in the CA1 region (AV axis −1.5 mm), where an equal volume of the AVV was injected at the same speed and dwell time as described above. Finally, the needle was withdrawn from the brain tissue at an extremely slow rate of 1 mm/min. This method minimizes brain tissue damage by using a single cranial entry point, while ensuring precise delivery and expression of the AAV in both target structures: CA1 and DG.

### 2.3 Epilepsy Models

In the acute PTZ model following viral intervention, 10 C57BL/6 mice (aged 6–8 weeks) per group received an intraperitoneal injection of PTZ (gamma‐aminobutyric acid receptor antagonist, 12.5 mg/kg, Sigma–Aldrich, USA) every 10 min for a total of eight injections. Seizure severity was assessed using the Racine grading scale [31]. When seizure scores reached Grade IV, the latency period to seizure onset and the number of injections required to induce tonic–clonic seizure were recorded. In the chronic PTZ model, a subthreshold dose of PTZ (35 mg/kg) was administered daily via intraperitoneal injection to 15 mice per group for 15 consecutive days [32]. The mice were observed for approximately 60 min after each injection, and seizure severity was assessed using the Racine scale. The chronic suprathreshold excitability model was considered successfully established if the mice exhibited Grade IV or V seizures for ≥ 3 consecutive days. The mice were anesthetized via intraperitoneal injection of a sterile pentobarbital sodium solution (Sigma, St. Louis, MO, USA; 50 mg/kg) and secured in a stereotactic frame (RWD Life Science, Shenzhen, China). A 0.5‐μL microsyringe was loaded with kainic acid (KA) solution. Stereotactic coordinates were set, using the Bregma point as the origin: AP, −2.0 mm; ML, ±1.5 mm; and DV, −2.0 mm. Microinfusion of KA was performed at 10 nL/min, and the needle was retained for 5 min after infusion to prevent reflux.

### 2.4 Behavioral Science and Field Potential Recording

Four groups of mice (15 per group) were established as KA‐induced epilepsy models, and behavioral changes between the experimental and control groups were analyzed through continuous video monitoring. Epilepsy susceptibility was assessed using the Racine scoring system. The primary outcome measures included the latency period to the first spontaneous seizure and the total number of SRS during the chronic phase (Days 14–28 after KA modeling). Specific methodologies have been described previously [33, 34].

A nickel–chromium alloy recording electrode (Kodo Brain Machine, Jiangsu, China) was precisely implanted into the right hippocampal CA1 region of the experimental mice using a stereotaxic instrument. The stereotaxic coordinates were calibrated with the anterior cranial fossa as the origin (AP: −2.0 mm; ML: −1.5 mm; DV: −1.5 mm). Following implantation, the electrode was secured with dental cement. Multichannel neurophysiological recording was performed to characterize field potential oscillations in the dorsal CA1 subregion of the hippocampus using nickel–chromium alloy electrodes after video monitoring. Electrophysiological signals were continuously acquired at a sampling rate of 500 Hz and were band‐pass filtered (1–100 Hz) to isolate oscillatory activities relevant to epileptiform discharge. The primary detection parameters included the cumulative count of epileptiform discharge events and their aggregate duration. A seizure‐like discharge event (SLE) was operationally defined as an abnormal electrical event satisfying three criteria: (1) spike–wave frequency exceeding 1 Hz, (2) waveform amplitude reaching at least twice the baseline level, and (3) single‐event duration surpassing 5 s; this result is consistent with established electrographic criteria for interictal and ictal‐like discharges [35, 36].

### 2.5 Quantification of Tissue Proteins

RIPA lysate (Beyotime Biotechnology, Shanghai, China) mixed with brain tissue was homogenized and ground. The homogenates were incubated on ice for 40 min to facilitate complete lysis, followed by centrifugation with 14,000 × *g* at 4°C for 15 min. The supernatants were carefully transferred to pre‐chilled sterile centrifuge tubes to avoid contamination and stored at −80°C until protein analysis.

Protein quantification was performed using the BCA Protein Assay Kit (Beyotime Biotechnology), according to the manufacturer's instructions. Based on the quantification results, appropriate volumes of a phosphate‐buffered saline (PBS) and 5 × sodium dodecyl sulfate–polyacrylamide gel electrophoresis (SDS‐PAGE) loading buffer were added to each sample to achieve consistent protein loading concentrations. After denaturation at 95°C for 10 min, the samples were aliquoted into microcentrifuge tubes and stored at −20°C to preserve protein integrity for subsequent SDS‐PAGE and western blot analyses.

### 2.6 Western Blot

Proteins were separated by 7.5% or 10% SDS‐PAGE (Aase Bio, Shanghai, China) and transferred to polyvinylidene difluoride membranes (Millipore, Bedford, MA, USA). The membranes were blocked with a protein‐free rapid blocking buffer (Aase Bio) for 25 min at room temperature, which was followed by overnight incubation with primary antibodies specific to the target proteins at 4°C for 14 h. After antigen–antibody binding, the membranes were washed three times with a TBST buffer (0.1% Tween‐20 in a Tris‐buffered saline solution; 5 min per wash). Horseradish peroxidase–conjugated secondary antibody (Proteintech, cat. SA00001‐2) was applied to the membranes at room temperature for 60 min, followed by repeated TBST washing (3 × 5 min). Protein signals were detected using a chemiluminescence imaging system. Target band grayscale values were quantified using image analysis software and normalized to internal reference GAPDH levels from the same sample. The following primary antibodies were used: rabbit anti‐TGM2 (1:1000, CST, cat. 3557S), rabbit anti‐PSD95 (1:100, CST, cat. 3450S), rabbit anti‐gephyrin (1:1000, Abcam, cat. ab228674), rabbit anti‐CD68 (1:1000, Proteintech, cat. 28,058–1‐AP), rabbit anti‐β‐actin (1:5000, Proteintech, cat. 66,009–1‐Ig), rabbit anti‐C1q‐A (1:1000, Santa Cruz Biotechnology, cat. sc‐58,920), rabbit anti‐C1q‐B (1:1000, ABclonal, cat. A5339), rabbit anti‐Tim‐4 (1:1000, Santa Cruz Biotechnology, cat. sc‐390,805), rabbit anti‐MerTK (1:1000, ABclonal, cat. A5443), and rabbit anti‐ABCA1 (1:1000, ABclonal, cat. A22125).

### 2.7 Proteomics

Tissue samples were ground in liquid nitrogen and subjected to ultrasonic extraction using lysis buffer containing 8 M urea and protease inhibitors. Protein concentration was determined using the BCA method. Equal volumes of protein solutions were precipitated with acetone and then trypsinized overnight in 200 mM TEAB. The proteins were reduced and alkylated prior to digestion. The resultant peptide fractions were separated using a NanoElute ultra‐high‐performance liquid chromatography system with mobile phases A (water containing 0.1% formic acid and 2% acetonitrile) and B (acetonitrile containing 0.1% formic acid) by following a gradient elution program. The separated peptides were ionized via a capillary ion source and analyzed on a timsTOF Pro 2 mass spectrometer using its data‐dependent acquisition (dda‐PASEF) mode.

### 2.8 Immunofluorescence Staining

Brain tissues were fixed in 4% paraformaldehyde at 4°C for 24 h, followed by sequential immersion in 15% and 30% sucrose solutions for cryoprotection (24–36 h each). The tissues were embedded in an optimal cutting temperature compound, and 15‐μm sections were created using a cryostat. Frozen sections were equilibrated at room temperature for 20 min, subjected to antigen retrieval by being microwaved in a sodium citrate buffer (high power for 3 min, low power for 10 min), and cooled to room temperature. The sections were permeabilized with 0.3% Triton X‐100 at 37°C for 30 min, blocked with goat serum (Sevier, Wuhan, China) at 37°C for 1 h, and incubated with primary antibodies at 4°C for 16 h. The primary antibody panel included: anti‐TGM2 (rabbit, Proteintech, cat. no. 15100‐1‐AP, 1:100), anti–glial fibrillary acidic protein (GFAP; rabbit, Proteintech, cat. no. 16825‐1‐AP, 1:100), anti–neuronal nuclei (NeuN; rabbit, Proteintech, cat. no. 16825‐1‐AP, 1:100), anti–ionized calcium‐binding adapter molecule (Iba‐1; rabbit, ZenBio, cat. no. 382207, 1:200), anti‐PSD95 (rabbit, CST, cat. no. 3450S, 1:200), anti‐CD68 (rabbit, Proteintech, cat. no. 28058‐1‐AP, 1:100), anti‐iNOS (rabbit, ZenBio, cat. no. 340668, 1:200), and anti‐ARG‐1 (rabbit, Proteintech, cat. no. 66129‐1‐AP, 1:800). After washing with PBS, the sections were incubated with a fluorescent secondary antibody (Aifang Bio, cat. no. AFIHC034; a universal, highly specific secondary antibody that has been specially engineered to simultaneously recognize primary antibodies from different species and enables multiplex labeling by coupling with different fluorescent dyes) at 37°C for 1 h in the dark. Finally, the sections were mounted with DAPI and visualized using laser confocal microscopy.

### 2.9 Golgi Staining

Following cardiac perfusion with the saline solution, the entire brain was rapidly dissected on ice and sectioned into thick coronal blocks. The blocks were immediately immersed in a pre‐chilled mixture containing 4% paraformaldehyde and 1% picric acid at 4°C under light‐protected conditions and gently rocked for 24 h. The tissues were then transferred into a fresh fixative and maintained at 4°C for 14 days; the fixative was replaced every 48 h to preserve efficacy. Following fixation, the tissue blocks underwent a two‐stage chromation process at 25°C in the dark using a 1% potassium dichromate solution (Solution C), with each stage lasting 36 h for a total of 72 h. The chromated tissue blocks were rinsed with distilled water, equilibrated in a −22°C freezer slider, and sectioned into 100 μm‐thick crown sections. The sections were rapidly frozen using pre‐chilled isopentane at −80°C, mounted onto gelatin‐coated slides, and dried in the dark at room temperature for 48 h. The dried sections were sequentially processed: stained in a D/E mixed staining solution (e.g., silver nitrate working solution) for 10 min under light‐protected conditions, dehydrated with graded ethanol solutions (50%, 70%, 85%, 95%, and 100%) for 4 min per concentration, and cleared with xylene for 4 min per treatment; this process was repeated three times. The sections were mounted with neutral resin and examined under an optical microscope. ImageJ was used to perform a quantitative analysis of dendritic spine density and morphology in specific brain regions based on standardized thresholds.

### 2.10 BV2 Microglia Culture

Cells were resuscitated, seeded in Dulbecco's Modified Eagle Medium/Ham's F‐12 (GIBCO, New York, USA) supplemented with 10% fetal bovine serum (Punosai, Wuhan, China), and maintained at 37°C in a 5% CO_2_ incubator. The culture medium was replaced every 48 h, and the cells were passaged using 0.25% trypsin digestion when 80%–90% confluence was achieved. A serum‐free medium was applied 24 h prior to intervention, and samples were collected at predefined time points after treatment.

### 2.11 Immunoprecipitation

A 50‐μL suspension of magnetic beads (MCE, Washington, USA) was washed twice with 400 μL PBST, and the supernatant was magnetically removed after each wash. Subsequently, 2 μg of anti‐ABCA1 antibody was added, and the mixture was incubated at 4°C on a rotary mixer for 4 h. The protein samples were quantified and split into Input and IP groups. Antibody‐coupled beads were magnetically separated, washed four times with PBST, and then added to the IP samples. The reaction proceeded overnight at 4°C on a rotary mixer. Following incubation, the beads were transferred into a new EP tube, washed four times with PBST, and resuspended in 65 μL of a 1× loading buffer. The samples were denatured by heating at 95°C for 5 min in a metal bath. The supernatant was collected for western blot analysis according to standard procedures.

### 2.12 Quantitative Real‐Time Polymerase Chain Reaction

Total RNA was extracted using an RNA extraction solution (Sewell Bio, cat. G3013) via a homogenization–chloroform substitution method, after which it was reverse transcribed into cDNA using the SweScript All‐in‐One RT SuperMix for qPCR Kit (Sewell Bio, cat. G3337). The real‐time quantitative polymerase chain reaction (qPCR) system comprised Universal Blue SYBR Green qPCR Master Mix (×2; Silver Biotech, cat. G3326), gene‐specific primers, and cDNA template, amplified on a Bio‐Rad CFX Connect qPCR instrument. Each sample was processed in triplicate, and *GAPDH* served as the internal control gene for normalization. All the qPCR primer sequences used are listed in Table S1. Relative gene expression levels were calculated using the 2^−ΔΔCT^ method.

### 2.13 Statistical Analysis

Data are presented as mean ± standard error of the mean, and all statistical analyses were conducted using GraphPad Prism v.10.1.2. The Shapiro–Wilk test was applied to validate the normal distribution of the data set. For evaluating differences between two datasets, an independent sample *t*‐test was conducted for data conforming to a normal distribution; data that deviated from a normal distribution were analyzed using the Mann–Whitney *U* test. For comparisons among multiple datasets, one‐way analysis of variance (ANOVA) and a Tukey's post hoc test were performed. *p* < 0.05 indicated statistical significance.

## 3 Results

### 3.1 TGM2 Expression in KA‐Induced Epileptic Mice

**FIGURE 1:**
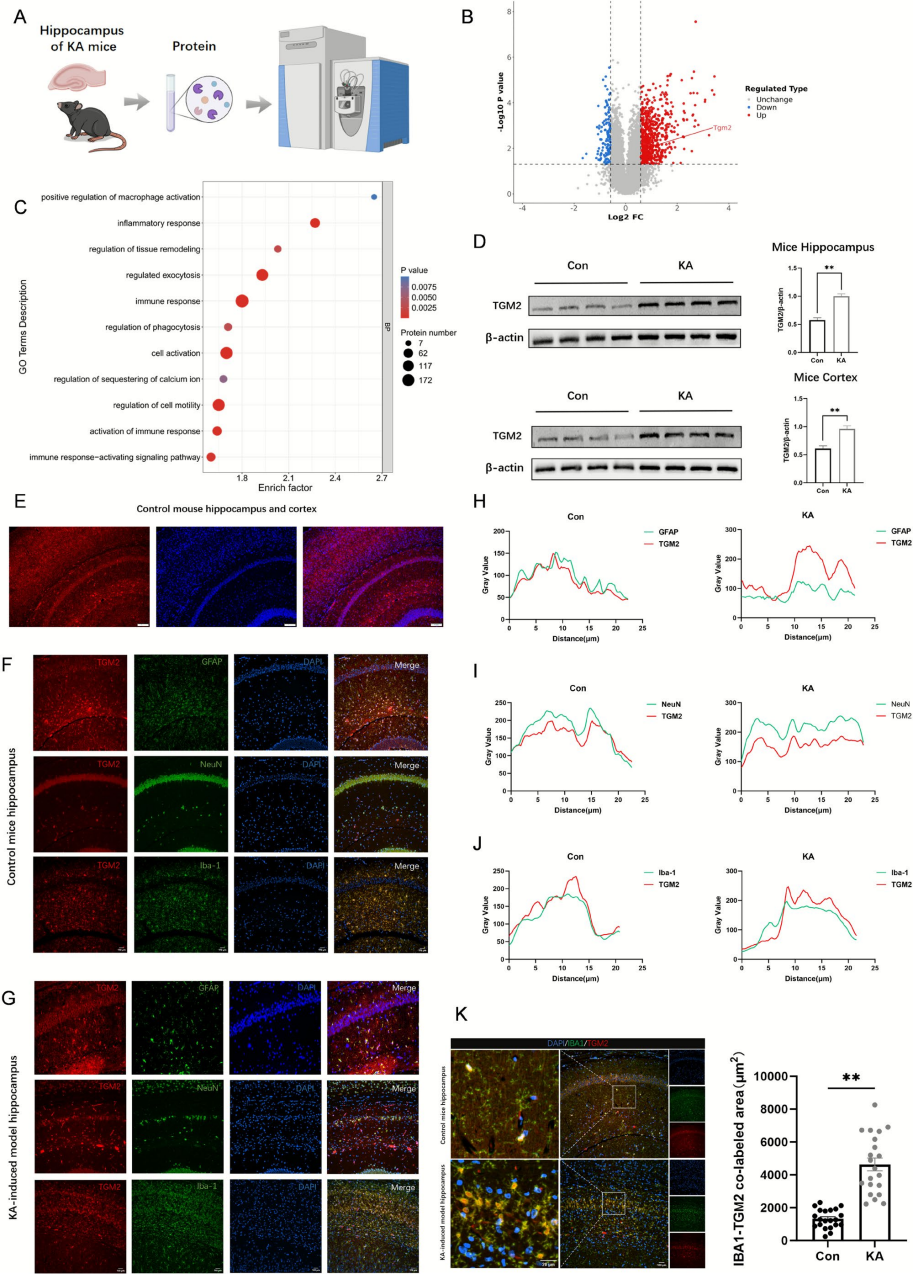
Expression and Localization of TGM2 in a Kainic Acid (KA)–Induced Epilepsy Mouse Model. (A) Schematic of sequencing in the hippocampus of epileptic mice. (B) Volcano plot of differentially expressed proteins in the hippocampus of epileptic mice (*p* < 0.05; |Log2FC| > 1.5, *n* = 6). (C) Bubble plots of GO–BP enrichment analysis of differentially expressed proteins in the hippocampus of epileptic mice (*n* = 6). (D) Differences in hippocampal and cortical TGM2 expression between the control (Con) and KA groups (**p < 0.01, nonparametric t‐test; *n* = 5). (E) Representative images depicting the TGM2 immunofluorescence staining of brain tissue (scale bar = 1 mm). Representative images of immunofluorescence staining for colocalization of TGM2 with GFAP, NeuN, and Iba1 in the hippocampal CA1 region of mice in the (F) Con group and (G) KA group (scale bar = 100 μm). (H–J) Fluorescence intensity ratios of TGM2 colocalized with GFAP, NeuN, and Iba1 in the hippocampus of mice in the KA and Con groups (*n* = 5). (K) Area ratio of TGM2 colocalized with Iba‐1 in the hippocampus of mice within the KA and Con groups (*n* = 3; **p < 0.01, nonparametric *t*‐test).

To investigate the biological function of TGM2 in the pathophysiology of epilepsy, a KA‐induced epilepsy model was developed and a whole‐proteome analysis of hippocampal tissues from six epileptic mice was performed (Figure 1A). Differential protein analysis revealed significantly elevated TGM2 protein levels (Figure 1B). Functional annotations indicated involvement of TGM2 in key biological processes, including positive regulation of phagocytosis, activation of immune responses, and tissue remodeling (Figure 1C). To explore the potential function of TGM2 in epileptogenesis, immunoblotting was performed to determine TGM2 expression levels in the KA‐induced epilepsy model. The hippocampal and temporal lobe tissues of the epileptic mice showed aberrantly high TGM2 protein levels compared with those of the control mice (*p* < 0.01) (Figure 1D).

In neural tissues, TGM2 localizes to the brain, spinal cord, and peripheral nerves [37]; however, its distribution in brain regions relevant to epilepsy (hippocampus and cortex) remains unclear. Immunohistochemistry of the cortex, CA1, CA3, and DG regions of the hippocampi was determined using NeuN, GFAP, and IBA1—specific markers of neurons, astrocytes, and microglia, respectively—to identify the cells expressing TGM2. The results demonstrated that TGM2 colocalized with GFAP, NeuN, and Iba‐1 (Figure 1F–J). Compared with the control mice, the epileptic mice exhibited increased TGM2 colocalization with microglia in the hippocampus (*p* < 0.01, Figure 1H). Collectively, these results demonstrated that the epilepsy model induced TGM2 overexpression and subsequent colocalization in microglia.

### 3.2 TGM2 Suppresses Seizure Susceptibility in KA and PTZ‐Induced Chronic Epilepsy

**FIGURE 2:**
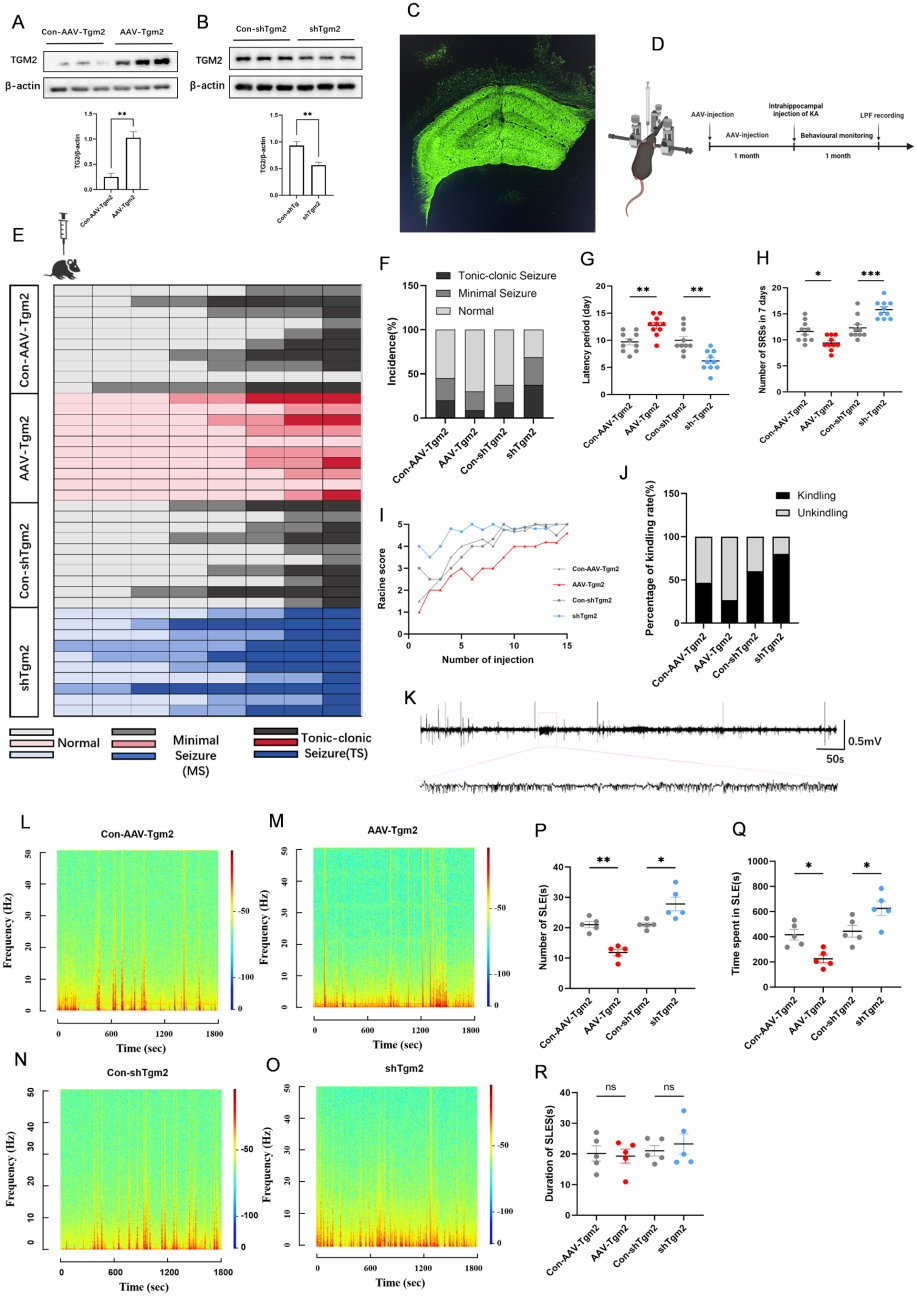
TGM2 modulates seizure susceptibility and severity. (A) Differences in TGM2 expression after AAV‐Tgm2 versus Con‐AAV‐Tgm2 injection (**p < 0.01, nonparametric t‐test; *n* = 5). (B) Differences in TGM2 expression after shTgm2 versus Con‐shTgm2 injection (**p < 0.01, non‐parametric *t*‐test; *n* = 5). (C) Viral injection sites and expression ranges of AAV‐Tgm2 or shTgm2. The figure illustrates precise fluorescence distribution in the hippocampal CA1 and dentate gyrus (DG) regions (scale bar = 100 μm). (D) Experimental design of the KA‐induced epilepsy model. (E) Heatmap of acute PTZ model progression (*n* = 10). (F) Seizure frequency in the acute PTZ mouse model (*p* < 0.01, Chi‐square and Fisher's exact tests; *n* = 10). (G) Seizure frequency in the last 7 days (one‐way ANOVA followed by Tukey's multiple comparison test; *n* = 10). (H) Behavioral monitoring showing seizure latency (*p < 0.05, ***p < 0.001; one‐way ANOVA followed by Tukey's multiple comparison test; *n* = 10). (I) Line graph of Racine grades in the chronic PTZ mouse model (*p* < 0.01, Chi‐square and Fisher's exact test; *n* = 15). (J) Seizure ignition rate in the Con‐AAV‐Tgm2, AAV‐Tgm2, Con‐shTgm2, and shTgm2 groups in the chronic PTZ mouse model (*p* < 0.01, Chi‐square and Fisher's exact test; *n* = 15). (K) Typical local field potential (LFP) recordings during spontaneous seizures in KA‐induced epileptic mice. (L–O) Spectrograms corresponding to Con‐AAV‐Tgm2, AAV‐Tgm2, Con‐shTgm2, and shTgm2 for each group of LFPs. (P) Total seizure duration, (Q) seizure frequency, and (R) mean seizure duration on LFP recordings (one‐way ANOVA followed by Tukey's multiple comparison test; *n* = 5).

To investigate the in vivo pathophysiological effects of TGM2 overexpression, the role of TGM2 in epilepsy susceptibility was evaluated using the KA‐induced epilepsy mouse model. Gain‐ and loss‐of‐function experiments were conducted by stereotactically injecting AAVs into the CA1 and DG regions of mice. The following AAVs were used: (1) AAV‐Tgm2 for TGM2 overexpression; (2) shTgm2 lentivirus for TGM2 knockdown; and (3) control vectors (Con‐AAV‐Tgm2 or Con‐shTgm2) expressing enhanced GFP (EGFP). Four weeks after AAV delivery, western blot analysis confirmed TGM2 overexpression/knockdown. Compared with Con‐AAV‐Tgm2, AAV‐Tgm2 significantly increased TGM2 expression (*p* < 0.01) (Figure 2A); meanwhile, shTgm2 significantly reduced TGM2 expression relative to Con‐shTgm2 (*p* < 0.01) (Figure 2B). Confocal microscopy visualized EGFP fluorescence in the hippocampus, confirming efficient viral infection (Figure 2C).

To determine whether the changes in TGM2 expression influenced the progression of chronic seizures, a KA‐induced chronic seizure model was established. Status epilepticus (SE) was alleviated after the administration of sodium pentobarbital, an anticonvulsant. The first spontaneous seizures typically occurred between 5 and 30 days (mean, 14 days) after sodium pentobarbital administration. To measure changes in spontaneous seizure dynamics during the chronic phase of epilepsy, chronic spontaneous seizures after KA injection were quantified. Behavioral monitoring—quantified using the Racine grading system—revealed that TGM2 overexpression significantly prolonged the latency period to first grade IV–V spontaneous seizures (*p* < 0.01) and decreased the frequency of grade ≥ IV seizures. Conversely, TGM2 knockdown shortened seizure latency (*p* < 0.01) and increased the frequency of convulsive seizures (*p* < 0.01) (Figure 2G,H).

Next the role of TGM2 in seizure susceptibility was evaluated in the mouse model. In the acute PTZ model, compared with Con‐AAV‐TGM2, AAV‐Tgm2 significantly reduced the incidence of tonic–clonic seizures (*p* < 0.05); however, no significant difference was observed in the frequency of minimal seizures. TGM2 knockdown induced the opposite effect (*p* < 0.01) (Figure 2E,F). In the chronic PTZ model, TGM2 overexpression alleviated the seizure severity as indicated by a decrease in Racine grade; meanwhile, TGM2 knockdown exacerbated the seizure severity, leading to an increased Racine grade (*p* < 0.01) (Figure 2I). Moreover, the epilepsy kindling rate was lower in the TGM2 overexpression group than in the control group, whereas TGM2 knockdown increased kindling rate (*p* < 0.01) (Figure 2J). These results demonstrate that modulating TGM2 expression significantly influences seizure susceptibility in PTZ‐treated mice.

Thirty days after KA treatment, multichannel local field potential recordings in the hippocampus of mice confirmed the electrophysiological seizure characteristics. Neuroelectrophysiological analysis showed that high‐frequency, recurrent SLEs occurred in all groups. Consistent with pre‐behavioral observations, the TGM2 overexpression group (AAV‐Tgm2) had a significantly lower rate of SLE events (*p* < 0.01) and a significantly shorter cumulative duration of epileptiform discharges (*p* < 0.05) when compared with the control group (Con‐AAV‐Tgm2) (Figure 2P,Q). The TGM2 knockdown group (shTgm2) exhibited significant increases in the duration of epileptiform discharges (*p* < 0.05) and frequency of SLE episodes when compared with the control group (Con‐shTgm2) (*p* < 0.01) (Figure 2P,Q). No statistically significant differences were observed in the mean duration of individual SLE episodes among the groups (*p* > 0.05) (Figure 2R). These results further support the notion that TGM2 suppresses seizure susceptibility and severity.

### 3.3 TGM2 Affects Hippocampal Dendritic Spines in the Epileptic Mouse Model

**FIGURE 3:**
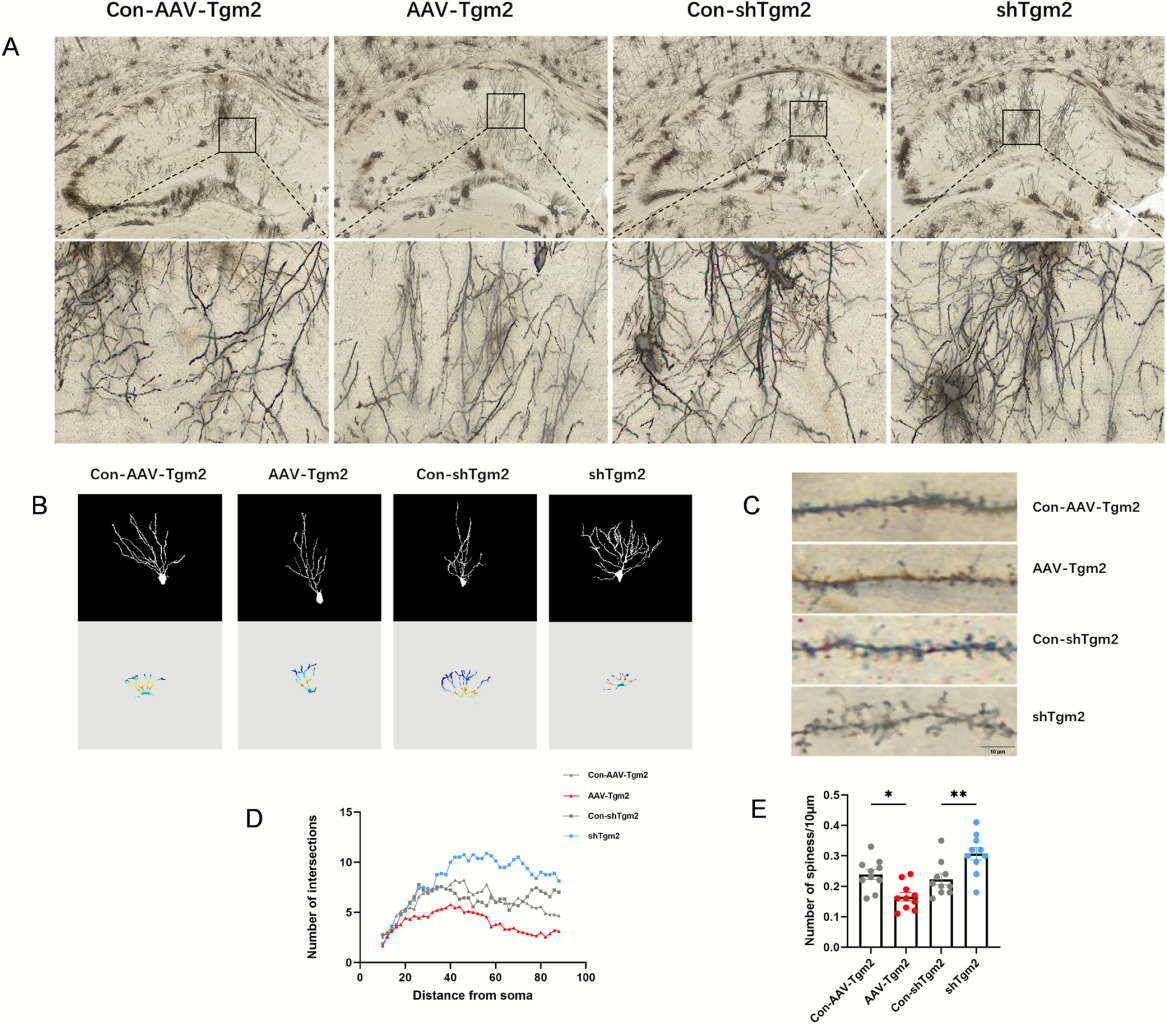
TGM2 regulates dendritic spine density. (A) Distribution of apical dendritic spines in the CA1 region of the hippocampus in the Con‐AAV‐Tgm2, AAV‐Tgm2, Con‐shTgm2, and shTgm2 groups, and local magnification of the dendritic spines (scale bar = 10; scale bar = 20). (B) Representative diagram of dendritic spine skeletons from hippocampal neurons of the four groups. (C) Schematic representing the oil microscopy of dendritic spines on the parietal dendrites in the CA1 hippocampal region of the four groups. (D) Sholl analysis of pyramidal neurons from the four groups corresponding to E. (E) Histogram of the density of dendritic spines on the parietal dendrites in the CA1 hippocampal region of the four groups. One‐way ANOVA is performed before conducting a Tukey's multiple comparison test. *p < 0.05 relative to Con‐AAV‐Tgm2; **p < 0.01 relative to Con‐shTgm2; *n* = 5.

Dendritic spines are small protrusions on neuronal dendrites. Multiple synapses can form on dendritic spines, which serve as crucial sites for neural signaling. Morphological changes in dendritic spines are often associated with synaptic plasticity [38]. In the present study, Golgi staining was performed to analyze the number and morphology of dendritic spines on the parietal dendrites in the CA1 hippocampal region in each experimental group of mice. Compared with the Con‐AAV‐Tgm2 group, the AAV‐Tgm2 group exhibited a significantly reduced number of dendritic spines (*p* < 0.05). Conversely, the shTgm2 group showed a significantly greater dendritic spine density compared with the Con‐shTgm2 group (*p* < 0.01) (Figure 3D,E).

### 3.4 Effect of TGM2 on Microglial Polarization

**FIGURE 4:**
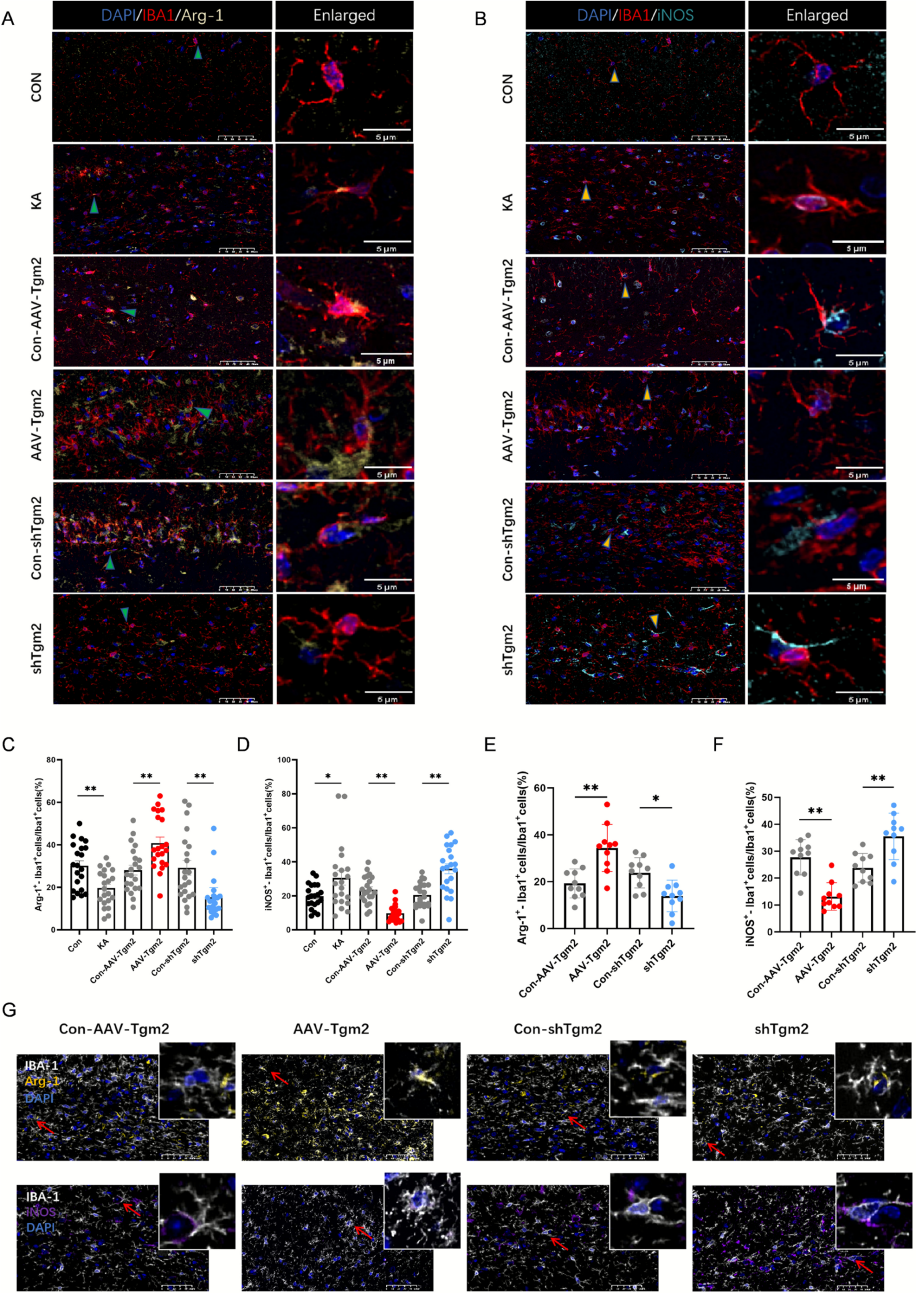
TGM2 modulates microglial polarization. Representative images of (A) Arg‐1^+^/Iba‐1^+^ and (B) iNOS^+^/Iba‐1^+^ immunofluorescence staining in the CA1 hippocampal region of mice in a kainic acid (KA)‐induced epilepsy model (scale bar = 100 μm). (C) Percentage of Arg‐1^+^/Iba‐1^+^ microglia in the hippocampus of each group in the KA epilepsy model (*n* = 21; one‐way ANOVA followed by Tukey's multiple comparison test; *p < 0.01 relative to the control; **p < 0.01 relative to Con‐AAV‐Tgm2; **p < 0.01 relative to Con‐shTgm2). (D) Percentage of iNOS^+^/Iba‐1^+^ microglia in the hippocampus of each group in the KA epilepsy model (*n* = 21). Percentage of (E) Arg‐1^+^/Iba‐1^+^ and (F) iNOS^+^/Iba‐1^+^ microglia in the hippocampus of each group in the PTZ‐induced chronic epilepsy model (*n* = 10). (G) Representative images of immunofluorescence staining for Arg‐1^+^/Iba‐1^+^ and iNOS^+^/Iba‐1^+^ in the CA1 hippocampal region in the PTZ‐induced chronic epilepsy model (scale bar = 100 μm).

Microglial activation is a critical event in epileptogenesis [39, 40]. Microglia polarize into two distinct phenotypes: M1 and M2. M1 microglia drive pro‐inflammatory responses, whereas M2 microglia mediate anti‐inflammatory events. Microglial activation in the KA‐induced chronic epilepsy model was assessed. To investigate whether TGM2 promotes microglial polarization, the protein expression levels of the proinflammatory marker iNOS and the anti‐inflammatory marker Arg‐1 in the CA1 hippocampal region were quantitatively analyzed. Immunofluorescence analysis showed that TGM2 overexpression increased the proportion of Arg‐1^+^ M2 microglia compared with the control treatment (*p* < 0.05), whereas TGM2 knockdown significantly decreased the proportion of Arg‐1^+^ microglia (*p* < 0.01) (Figure 4A,C). The proportion of iNOS^+^/Iba‐1^+^ M1 microglia was increased following TGM2 knockdown (*p* < 0.01) and decreased after TGM2 overexpression (*p* < 0.05) (Figure 4B,D). In the PTZ‐induced chronic epilepsy model, microglial polarization in the hippocampus followed a similar pattern to that observed in the KA‐induced chronic epilepsy model (Figure 4E–G). This observation demonstrates that TGM2 modulates microglial polarization, promoting microglial transition toward the M2 phenotype. This finding is consistent with prior investigations highlighting TGM2 as a specific marker for the M2 microglia phenotype [41] and showing that cystamine—a TGM2 inhibitor—significantly attenuates microglia‐mediated Aβ uptake in neuronal/glial mixed cultures [42].

### 3.5 Effect of TGM2 on the Microglial Phagocytosis of Synapses

**FIGURE 5:**
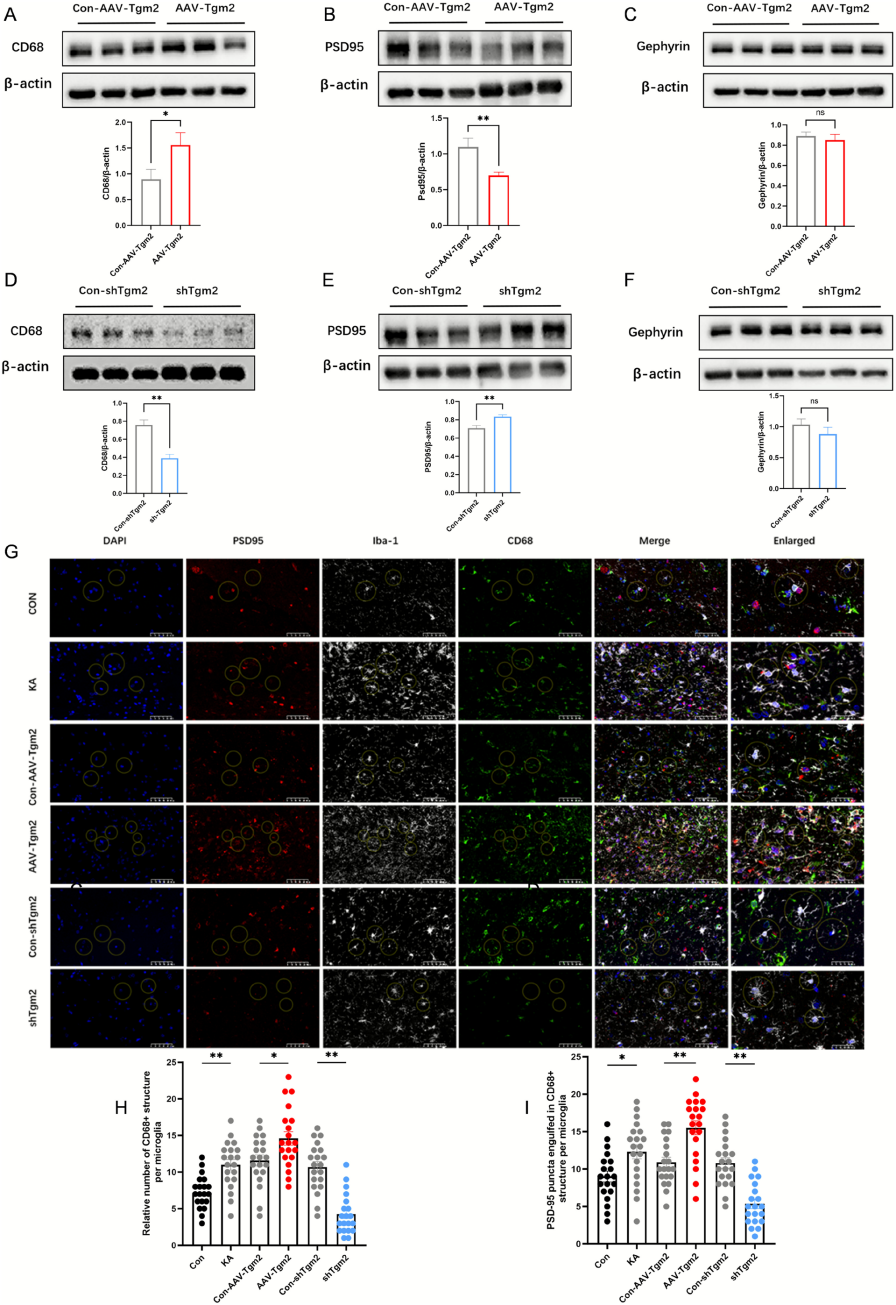
TGM2 regulates microglial phagocytosis of synapses. (A) CD68, (B) PSD95, and (C) gephyrin expression in the hippocampus of Con‐AAV‐Tgm2 and AAV‐Tgm2 mice in the epilepsy model (*p < 0.05, **p < 0.01, ns > 0.05; nonparametric t‐test; *n* = 5 each). (D) CD68, (E) PSD95, and (F) gephyrin expression in the hippocampus of Con‐shTgm2 and shTgm2 mice in the epilepsy model (*n* = 5 each). (G) Representative immunofluorescence images of microglia colocalized with CD68/PSD95 (scale bar = 50 μm). All histological data were collected from the CA1 hippocampal region. (H) Quantification of the number of CD68^+^ structures in microglia (*n* = 20; one‐way ANOVA followed by Tukey's multiple comparison test; **p < 0.01 relative to control; **p < 0.05 relative to Con‐AAV‐Tgm2; **p < 0.01 relative to Con‐shTgm2). (I) Quantification of the number of PSD95 puncta phagocytosed by microglia within CD68^+^ structures (*n* = 20).

CD68 expression reflects microglial phagocytic capacity, and microglia mediate synaptic pruning by engulfing redundant synapses; therefore, western blot analysis was conducted on hippocampal tissues isolated from AAV‐Tgm2‐injected and shTgm2‐injected mice 1 month after SE induction. Compared with the Con‐AAV‐Tgm2 group, the AAV‐Tgm2 group showed increased CD68 protein expression and decreased PSD95 expression in the hippocampus (*p* < 0.05) (Figure 5A,B). Conversely, the shTgm2 group exhibited reduced CD68 and increased PSD95 expression relative to the Con‐shTgm2 group (*p* < 0.01) (Figure 5D,E). Gephyrin expression remained unchanged in all groups (*p* > 0.05) (Figure 5C,F).

Immunofluorescence analysis of the CA1 hippocampal region further revealed that compared with the control and Con‐AAV‐Tgm2 groups, the AAV‐Tgm2 group exhibited a significantly enhanced mean CD68^+^ fluorescence intensity within microglia at the single‐cell level. Similarly, the number of PSD95 spots colocalized with CD68 around individual microglia was markedly increased (*p* < 0.01) (Figure 5G,H). In contrast, the shTgm2 group exhibited a reduced mean CD68^+^ fluorescence intensity per glial cell and a significant decrease in the number of PSD95–CD68 colocalization sites per cell (*p* < 0.01) (Figure 5G,I). Collectively, these findings indicate that TGM2 enhances the phagocytic functions of microglia, promoting the selective phagocytosis of excitatory synapses.

### 3.6 TGM2 Regulates the Expression of Proteins Involved in Synaptic Phagocytosis

**FIGURE 6:**
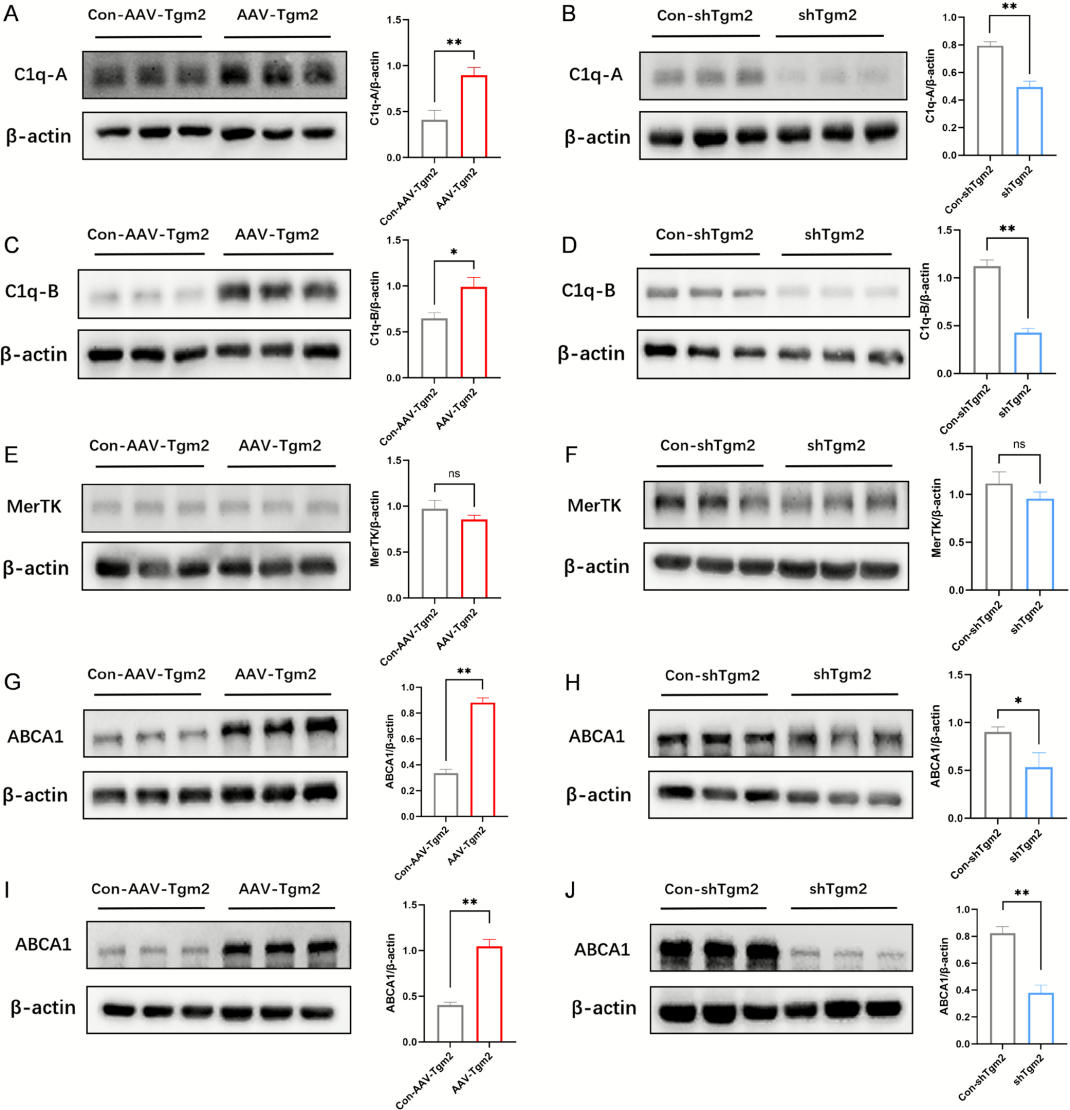
TGM2 regulates the expression of proteins involved in synaptic phagocytosis. (A, C, E, and G) Hippocampal C1q‐A, C1q‐B, MerTK, and ABCA1 expression in Con‐AAV‐Tgm2 vs. AAV‐Tgm2 mice in the kainic acid (KA) epilepsy model (**p < 0.01; *p < 0.05; ns > 0.05, non‐parametric *t*‐test; *n* = 5). (B, D, F, and H) Hippocampal C1q‐A, C1q‐B, MerTK, and ABCA1 expression in Con‐shTgm2 vs. shTgm2 mice in the KA epilepsy model (*n* = 5). (I) Comparative expression of ABCA1 in the hippocampal region of Con‐AAV‐Tgm2 and AAV‐Tgm2 mice in the PTZ‐induced chronic epilepsy model (*n* = 5). (J) Comparative expression of ABCA1 in the hippocampal region of Con‐shTgm2 and shTgm2 mice in the PTZ‐induced chronic epilepsy model (*n* = 5).

Key phagocytic proteins, including C1q‐A, C1q‐B, ABCA1, and MER proto‐oncogene tyrosine kinase (MerTK), were analyzed. The hippocampal expression levels of C1q‐A, C1q‐B, and ABCA1 were significantly elevated in the AAV‐Tgm2 mice compared with those in the Con‐AAV‐Tgm2 mice (*p* < 0.05) (Figure 6A,C,G). Conversely, the shTgm2 mice showed reduced expression of C1q‐A, C1q‐B, and ABCA1 compared with the Con‐shTgm2 mice (*p* < 0.05) (Figure 6B,D,H). MerTK expression did not significantly differ between the groups (*p* > 0.05) (Figure 6E,F). Compared with the control mice, increased ABCA1 expression was measured in the mice with PTZ‐induced chronic epilepsy following TGM2 overexpression, whereas decreased ABCA1 expression was measured following TGM2 knockdown (*p* < 0.01) (Figure 6I,J).

### 3.7 TGM2 Inhibits ABCA1 Ubiquitination

**FIGURE 7:**
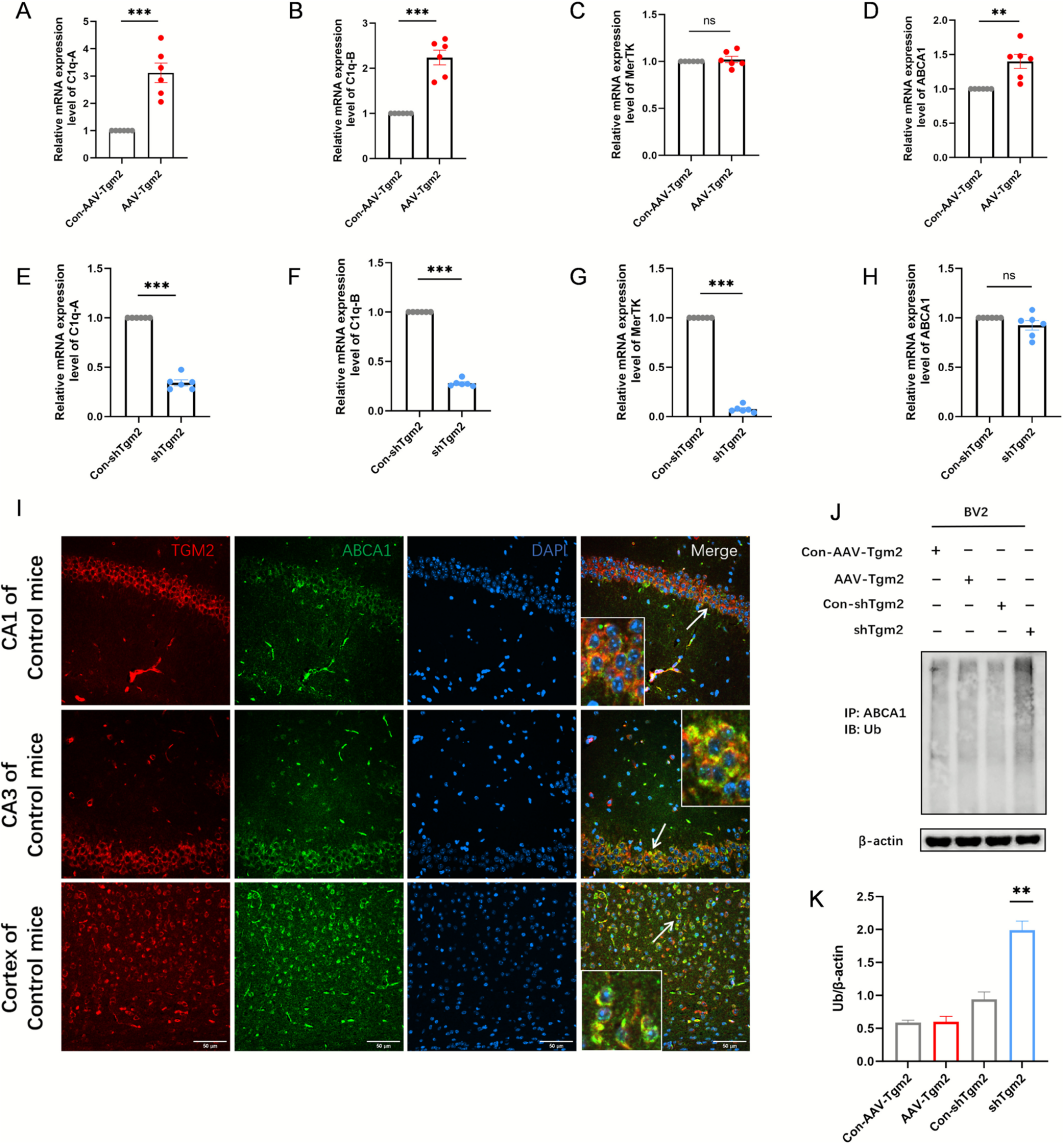
Mechanisms underlying TGM2‐mediated synaptic phagocytosis. (A–H) The relative mRNA expression levels of genes associated with phagocytosis in microglia were analyzed using quantitative real‐time polymerase chain reaction (***p < 0.01, non‐parametric *t*‐test; *n* = 6). (I) Representative images of immunofluorescence staining for colocalization of TGM2 with ABCA1 in the CA1, CA3, and cortex regions of the hippocampus of mice in the control group (scale bar = 50 μm). (J) ABCA1 protein ubiquitination level. (K) In the BV2 microglia cell line, the shTgm2 group exhibited significantly different ubiquitin expression compared with the Con‐AAV‐Tgm2, AAV‐Tgm2, and Con‐shTgm2 groups (**p < 0.01; one‐way ANOVA followed by Tukey's multiple comparison test; *n* = 5).

PCR analysis revealed that TGM2 expression levels influenced the gene transcription of multiple synaptic phagocytosis markers. Specifically, TGM2 knockdown significantly reduced the expression of C1q‐A, C1q‐B, and MerTK (*p* < 0.001) (Figure 7E–G), whereas no significant change was observed in ABCA1 expression (*p* > 0.05) (Figure 7H). TGM2 overexpression upregulated C1qa, C1qb, and ABCA1 expression (*p* < 0.01) (Figure 7A,B,D), whereas no significant change in MerTK expression was observed (*p* > 0.05) (Figure 7C). Collectively, these data indicate that TGM2 plays a crucial role in promoting synaptic phagocytosis. Given that TGM2 maintains protein stability by inhibiting protein degradation via TRIM21‐mediated ubiquitination following GTP binding [43] and that ABCA1 is degraded through ubiquitination [44, 45, 46], we identified TGM2 and ABCA1 colocalization in the brain tissue of the control mice via immunofluorescence (Figure 7I). Subsequently, we explored whether TGM2 regulates ABCA1 ubiquitination. Following AVV intervention in the microglial cell line BV2, ABCA1 was immunoprecipitated from BV2 cells using an anti‐ABCA1 antibody. Ubiquitination levels were detected via western blotting with an anti‐ubiquitin antibody. TGM2 knockdown significantly enhanced ABCA1 ubiquitination and degradation (Figure 7J,K), indicating that TGM2 stabilizes ABCA1 protein levels by inhibiting its ubiquitination.

## 4 Discussion

The pathological mechanism of epilepsy—a complex neurological disease—involves abnormal neuronal discharge, synaptic remodeling, and glial cell activation. In recent years, the role of microglia in epileptogenesis has attracted attention. As immune cells of the CNS, microglia play a crucial role in maintaining the homeostasis and health of the brain. During seizures, microglia can be activated and undergo a series of morphological, functional, and associated molecular expression changes [47], which are closely related to the occurrence, development, and prognosis of epilepsy [48]. The present study revealed, for the first time, the bidirectional regulatory role of TGM2 in epileptogenesis and the potential mechanism by which TGM2 mediates synaptic remodeling through microglia.

In mouse models of epilepsy, the current study measured significantly elevated TGM2 expression levels in the hippocampus and cortex and showed enhanced colocalization of TGM2 with microglia in hippocampal regions. These key findings suggest that TGM2 may regulate the microglial functions involved in the pathological progression of epilepsy. TGM2 is a calcium‐dependent acyltransferase, and previous studies have demonstrated its involvement in apoptosis [43], inflammatory responses [49], and protein cross‐linking [50]; however, its regulatory role in glial cell function in epilepsy was previously unknown. In this experiment, we found that TGM2 expression was significantly and negatively correlated with the latency period and frequency of SRS. Specifically, viral‐mediated TGM2 overexpression (AAV‐Tgm2) and knockdown (shTgm2) models were established, and TGM2 overexpression prolonged the latency period and reduced the frequency of seizures, whereas TGM2 knockdown exacerbated epilepsy progression. This phenomenon suggests that TGM2 may inhibit epileptogenesis through an endogenous protective mechanism.

Dendritic spine density is closely related to epilepsy onset and development; its aberrant changes are involved in the mechanisms of epilepsy by impacting the efficiency of synaptic connectivity, neural network excitability, and pathological remodeling [51, 52, 53]. In the current epilepsy model, Golgi staining revealed reduced dendritic spine density following TGM2. TGM2 may affect neural networks through a dual mechanism, which enhances the clearance of aberrant synapses by microglia [9, 54] and directly or indirectly inhibits synaptic overgrowth [55]. Moreover, TGM2 overexpression ultimately improved the seizure condition, suggesting that moderate synaptic pruning may exert a protective effect by eliminating abnormal neural circuits.

Subsequent investigations focused on the mechanism by which TGM2 regulates microglial phenotype shifts and phagocytic function. Microglia exhibit high plasticity, and the balance between their pro‐inflammatory (M1; marker, iNOS) and anti‐inflammatory (M2; marker, Arg‐1) phenotypes is crucial for maintaining homeostasis in neuroinflammatory and synaptic processes [56, 57]. The present study found that TGM2 overexpression significantly increased the proportion of Arg‐1+/Iba‐1+ M2 microglia, while reducing the proportion of iNOS+ M1 microglia; TGM2 knockdown showed the opposite trend, indicating a shift toward a pro‐inflammatory state. These results indicate that TGM2 is a key driver of microglial polarization toward the M2 phenotype. Notably, within the M2 spectrum, the M2a subtype—which is particularly induced by interleukin (IL)–4/IL‐13 signaling—has been renowned for its potent neuroprotective and reparative functions [58, 59]. M2a microglia secrete neurotrophic factors, such as insulin‐like growth factor‐1, and upregulates IL‐10 expression to establish an anti‐inflammatory microenvironment. These characteristics align well with the observed improvements in the epileptic condition and synaptic remodeling in the current study. Therefore, we hypothesize that TGM2 may preferentially promote microglia polarization toward the M2a subtype by potentiating cytokine signaling, such as IL‐4/IL‐13 signaling. Meanwhile, the enzymatic activity and scaffold protein functions of TGM2 may directly enhance the phagocytic capacity of microglia. This dual effect of M2a polarization (creating a reparative environment) and enhanced phagocytosis (clearing aberrant synapses) mediates the neuroprotective effects of TGM2 [50, 58]. Future studies should verify whether TGM2 specifically regulates M2a‐associated markers, thereby refining this mechanistic pathway.

TGM2 selectively regulates microglial phagocytosis of excitatory synapses without significantly affecting inhibitory synapse phagocytosis; this result may be related to the variations in molecular mechanisms by which different synapse types are recognized. Studies have confirmed that C1q and C3 molecules in the complement system—part of the innate immune system—specifically recognize the structural components of excitatory synapses phagocytosed by microglia. In the classical activation pathway, cascade activation of the complement system is triggered by C1q molecules, which in turn mediate the activation of C3 and its downstream receptors; this ultimately regulates the clearance of synaptic components through specific signaling pathways [60]. However, the phagocytosis of inhibitory synapses may be influenced by other regulatory pathways regardless of the complement system. A previous experiment verified that inhibitory synapse pruning by microglia is selectively regulated by GABABRs [61]. Therefore, the mechanism associated with selective clearance of excitatory synapses is closely related to the functional state of the complement system. The findings of the current experiment support this biological relationship, such that TGM2 overexpression significantly promotes the synthesis of C1q‐A/B isoforms, suggesting that TGM2 may play a role in promoting excitatory synapse modification in microglia by modulating phagocytosis via the complement cascade.

The ABCA1 protein exhibits a wide range of tissue expression properties in the human body, and its distribution characteristics are closely related to the physiological activities of cholesterol metabolism. At the organ level, ABCA1 showed abundant expression in liver parenchymal cells, alveolar epithelial cells, and the CNS. In the immune system, ABCA1 was found to be highly concentrated in macrophage populations in inflammation‐associated immune cells, as well as in the mononuclear cell lineage [62, 63, 64]. The molecular structure of ABCA1 consists of two transmembrane domains (TMDs) and two intracellular nucleotide‐binding domains (NBDs): the TMDs form hydrophobic channels that specifically bind cholesterol and phospholipids, whereas the NBDs provide energy to drive lipid efflux via ATP hydrolysis [65]. The selection of ABCA1 as a target for ubiquitination studies is mainly based on its dual properties: (1) ABCA1 affects the membrane localization of phagocytic receptors by regulating the lipid raft structure [66, 67]; (2) its cholesterol‐transporting function has been closely associated with the activation of the complement protein C1q, which may promote the assembly of C1q complexes by maintaining cholesterol homeostasis in the cell membrane [68]. The current experiment showed that the changes in ABCA1 expression were synchronized with the levels of C1q‐B. This suggests that ABCA1 may act as a pivotal molecule regulating both complement labeling and phospholipid receptor–mediated synaptic recognition by remodeling the lipid microenvironment.

TGM2 is involved in the regulation of ABCA1. Herein, TGM2 overexpression increased both the mRNA and protein levels of ABCA1, suggesting that TGM2 may positively regulate ABCA1 expression at the transcriptional level by activating relevant transcription factors or signaling pathways [24, 69, 70]. Concurrently, ABCA1 ubiquitination levels were unchanged, suggesting that TGM2 overexpression may counteract degradation pathways by stabilizing the ABCA1 protein, which is realized by inhibiting ubiquitination or promoting deubiquitination processes. Conversely, TGM2 knockdown was associated with decreased ABCA1 protein levels and increased ABCA1 ubiquitination, while ABCA1 mRNA levels remained unchanged. These findings strongly indicate that TGM2 deficiency primarily impacts post‐translational stability: endogenous TGM2 likely protects ABCA1 from ubiquitination via its enzymatic activity or other interactions. Following knockdown, the protection offered by TGM2 is lost, leading to enhanced ABCA1 ubiquitination and accelerated degradation. TGM2 regulates ubiquitination processes through a specific molecular mechanism: TGM2 activity is dependent on GTP binding and can effectively antagonize the TRIM21‐dependent ubiquitination process, thus promoting the maintenance of target protein homeostasis [43]. Additionally, studies have shown that TRIM21 overexpression in macrophages significantly reduces the protein expression of ABCA1, suggesting a functional regulatory relationship between the two proteins [71]. The present study confirmed that TGM2 modulates ABCA1 ubiquitination. We hypothesized that TGM2 may mediate ABCA1 ubiquitination by inhibiting TRIM21, thus increasing ABCA1 protein stability and the expression level of its related protein C1q‐B.

Although the present study established TGM2 as a critical regulator of microglial function and synaptic remodeling in epilepsy, several questions remain. The specific molecular pathways by which TGM2 regulates ABCA1 ubiquitination, including TGM2–TRIM21 interaction dynamics, require clarification. Additionally, microglia‐specific TGM2 knockdown models lack validation. This limitation implies that although our current data strongly support TGM2 as a key regulator of the neuroinflammatory microenvironment, the in vivo functions of TGM2 cannot be entirely attributed to microglia‐derived TGM2 alone. The complexity of the in vivo environment suggests that microglial TGM2 likely functions within a more intricate cellular network. Future investigations could employ single‐cell sequencing to characterize the microglial subpopulations affected by TGM2 and integrate electrophysiological techniques to explore synaptic transmission changes, thus delineating the mechanistic role of TGM2 in epileptogenesis.

## 5 Conclusion

TGM2 was revealed as an intrinsic neuroprotective factor in epilepsy. Functionally, TGM2 expression levels inversely correlated with epilepsy severity. At the cellular level, TGM2 synergistically improved neuroinflammation and synaptic imbalance by promoting M2 microglia polarization and enhancing the capacity of microglia to clear excitatory synapses. At the molecular level, TGM2 function depends on its regulation of the complement pathway and the stability of the ABCA1 protein. Our work not only clarifies the central role of TGM2 in the multidimensional mechanisms of epileptogenesis but also establishes a theoretical foundation for its potential as a therapeutic target. In summary, our findings highlight TGM2 as a novel mediator of microglia‐dependent synaptic remodeling, offering new insights into epileptic pathogenesis and potential therapeutic targets.

## Author Contributions

Z.Z. and X.W. contributed equally to this work. H.Z.: conceptualization. Z.Z., J.Q., and Q.Q.: formal analysis. B.F., Q.Q., and X.W.: data curation. Z.Z. and Y.X: manuscript editing. Z.Z. and J.Q.: writing – original draft preparation. H.Z., Z.X., and J.Y.: writing – review and editing. J.Y., X.X., and J.L.: supervision. J.T., L.Z., and H.H.: project administration. H.Z., Z.X., and C.Y.: funding acquisition. All authors have read and approved the final version of this manuscript and agree to be accountable for all aspects of the work.

## Funding

This work was supported by the National Natural Science Foundation of China (32460197; 32160190; and 82171440), Natural Science Foundation of Guizhou (grant number is QKHJC‐ZK[2025]‐401), and the Natural Science Foundation of Chongqing (CSTB2024NSCQ‐MSX0027).

## Ethics Statement

All animal experiments in this study strictly adhered to the relevant ethical guidelines for laboratory animal welfare. The research protocol was reviewed and approved by the Ethics Committee of Zunyi Medical University Affiliated Hospital (Approval No.: zyfy‐an‐2023‐0244) and the Animal Care and Use Committee of Chongqing Medical University (Approval No.: IACUC‐CQMU‐2024‐0277).

## Conflicts of Interest

The authors declare no conflicts of interest.

## Supporting information


**Table S1:** All primers used for qPCR.

## Data Availability

The data that support the findings of this study are available from the corresponding author upon reasonable request.
